# Enhancing Nitrogen Reduction Reaction through Formation of 2*D*/2D Hybrid Heterostructures of MoS_2_@rGO

**DOI:** 10.1021/acsami.4c00719

**Published:** 2024-04-30

**Authors:** Joyce B. Matsoso, Nikolas Antonatos, Lukáš Dekanovský, Roussin Lontio Fomekong, Joshua D. Elliot, Diego Gianolio, Vlastimil Mazánek, Catherine Journet, Zdeněk Sofer

**Affiliations:** † Department of Inorganic Chemistry, University of Chemistry and Technology in Prague, Technická 5, 166 28 Prague 6, Czech Republic; ‡ Laboratoire des Multimatériaux et Interfaces, UMR CNRS 5615, Univ-Lyon, Université Claude Bernard Lyon 1, F-69622 Villeurbanne, Cedex, France; § Department of Semiconductor Materials Engineering, Faculty of Fundamental Problems of Technology, Wrocław University of Science and Technology, Wybrzeże Wyspiańskiego 27, 50-370 Wrocław, Poland; ∥ Diamond Light Source, Diamond House, Harwell Science and Innovation Park, Didcot OX11 0DE, Oxfordshire, U.K.

**Keywords:** 2D/2D heterojunction hybrid, rGO, MoS_2_, interface, alkaline media, nitrogen reduction reaction

## Abstract

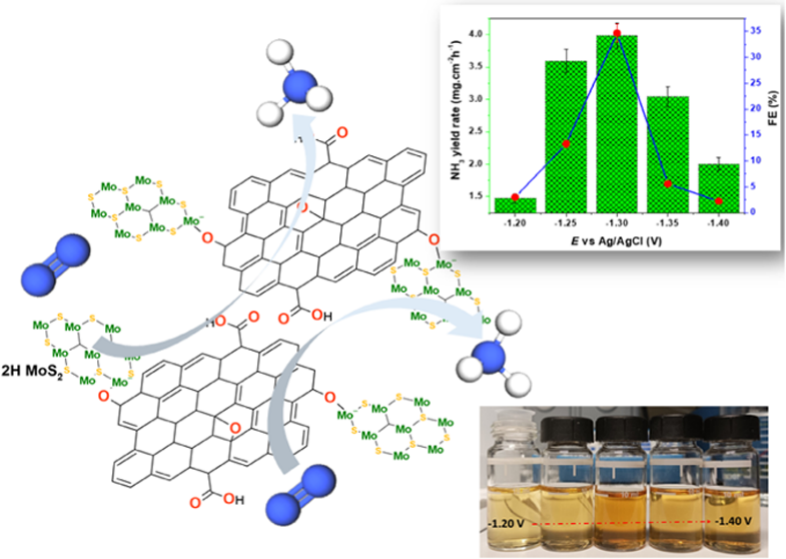

Given the challenging task of constructing an efficient nitrogen reduction reaction (NRR) electrocatalyst with enhanced ambient condition performance, properties such as high specific surface area, fast electron transfer, and design of the catalyst surface constitute a group of key factors to be taken into consideration to guarantee outstanding catalytic performance and durability. Thereof, this work investigates the contribution of the 2D/2D heterojunction interface between MoS_2_ and reduced graphene oxide (rGO) on the electrocatalytic synthesis of NH_3_ in an alkaline media. The results revealed remarkable NRR performance on the MoS_2_@rGO 2*D*/2D hybrid electrocatalyst, characterized by a high NRR sensitivity (faradaic efficiency) of 34.7% with an NH_3_ yield rate of 3.98 ± 0.19 mg h^–1^ cm^–2^ at an overpotential of −0.3 V vs RHE in 0.1 M KOH solution. The hybrid electrocatalysts also exhibited selectivity for NH_3_ synthesis against the production of the hydrazine (N_2_H_4_) byproduct, hindrance of the competitive hydrogen evolution reaction (HER), and good durability over an operation period of 8 h. In hindsight, the study presented a low-cost and highly efficient catalyst design for achieving enhanced ammonia synthesis in alkaline media via the formation of defect-rich ultrathin MoS_2_@rGO nanostructures, consisting predominantly of an HER-hindering hexagonal 2H-MoS_2_ phase.

## 1 Introduction

Clean and sustainable ammonia (NH_3_) is considered an ideal carbon-free energy carrier useful for mitigating issues related to environmental pollution as well as drastic consumption of nonrenewable fossil fuels during the production of inorganic chemicals for the agricultural, medical, or pharmaceutical industries.^1,2^ In industry, NH_3_ production depends predominantly on the century-old Haber–Bosch process that operates at high temperature and pressure (∼500 °C, ∼25 MPa) over iron (Fe)- and/or ruthenium (Ru)-based catalysts, consuming ∼2% of the global energy and subsequently generating up to 2 tons of yearly worldwide greenhouse gas emissions.^3^ For this reason, it is strikingly important that the development of greener, more energy-efficient, and sustainable routes to produce NH_3_ is investigated and employed. To date, various methods such as the biological method using purified nitrogenase bacteria^4^ and the photocatalysis^5^ and electrocatalysis^6,7^ routes have been explored to promote the reduction of dinitrogen (N_2_) to NH_3_ at ambient conditions. Among these reduction routes, the electrocatalytic nitrogen reduction reaction (NRR, N_2_ + 6H^+^ + 6e^–^ →2NH_3_) has emerged as a promising, environmentally benign, and sustainable technology for N_2_ fixation due to its mild operation conditions, utilizing water as a clean raw material, and no greenhouse gas (CO_2_) emission.^1,2,8^ Ideally, the best NRR process is characterized by a catalyst with a small activation energy (Ea) to N_2_ but also having relatively weak adsorption energy (Δ*E*) for intermediate species. Nonetheless, the electrocatalytic NRR is far from being used widely in practical applications owing to the fact that most of the currently used electrocatalysts display poor NRR kinetics and low faradaic efficiency (FE).^2,9^ The unfavorable N_2_ adsorption and activation, as well as the parasitic hydrogen evolution reaction (HER) on the electrocatalysts, largely contribute to the impractical large-scale application of the NRR process.^1,9,10^ As a consequence, highly efficient electrocatalysts that can accelerate NRR kinetics and promote better faradaic efficiency are pursued.

Recently, various noble metal electrocatalysts (Au, Ag, Ru, Rh) have been used for electrochemical N_2_ fixation; however, their low abundance and high cost hinder their widespread usage in electrocatalytic NRR processes.^11,12^ To bridge the gap between economic feasibility and NRR catalytic efficiency, intensive efforts have been devoted to designing and developing nonprecious metal alternative NRR electrocatalysts, such as metal oxides (Fe_2_O_3_, TiO_2_, InO_2_, MoO_3_, etc.), transition-metal chalcogenides, MXenes, as well as metal-free materials (B_4_C, graphene, black phosphorus, graphitic carbon nitride (gC_3_N_4_)).^6,13−15^ Among these materials, the low-cost 2D molybdenum disulfide (MoS_2_) nanostructures have been pursued as potential NRR catalysts owing to their nontoxicity, high chemical stability in liquid media, and poor activity for competitive hydrogen evolution reaction (HER).^14,16,17^ Regardless of the great efforts devoted to optimizing the performance of MoS_2_-based NRR electrocatalysts, poor electrical conductivity, unsatisfactory NH_3_ yield, and low FE values have not been encouraging factors for the large-scale application of MoS_2_ nanostructures.^16,18−20^

To mitigate this issue, studies have shown that catalyst engineering through the formation of MoS_2_ 2*D*/2D heterojunction hybrid structures with other 2D materials such as graphene, gC_3_N_4_, and phosphorene can significantly improve NRR activity and durability.^21−23^ In general, using 2*D*/2D heterojunction structures for the NRR process is largely beneficial due to the potentially increased active sites, the large interface contact areas, and strong interface interactions between the two 2D materials, thereby promoting interfacial electronic coupling and charge transfer.^14,20,22−25^ Among the various 2D materials, graphene (Gr) or its derivatives is the most popular material to be used as a scaffold for MoS_2_ nanosheets due to its large surface area, superior electrical conductivity, high charge mobility, and intrinsic flexibility.^26,27^ For instance, Wu et al. showed that the MoS_2_ nanosheet-reduced graphene oxide hybrid (MoS_2_-rGO) electrocatalyst achieved an FE of 4.58% and a high NH_3_ yield rate of 24.82 μg h^–1^ mg_cat_.^–1^ as well as effectively suppressed HER.^23^ Despite the low FE, they attributed the NRR performance to abundant high-speed electron transport channels and the synchronously induced electronic coupling effect. As such, this study aims at increasing the NRR electrocatalyst performance by shifting the chemical equilibrium for HER by decreasing the concentration of the proton donor species (H_2_O or H_3_O^+^), through an increase in electrolyte pH, while also focusing on the contribution of the MoS_2_ phases on the NRR activity.

## 2 Experimental Section

### 2.1 Synthesis of MoS_2_@rGO Nanostructures

The MoS_2_@rGO composite nanostructures were synthesized by using the hydrothermal technique. Initially, the reduced graphene oxide (rGO) nanosheets were synthesized via the microwave-assisted reduction procedure as previously reported.^28^ Typically, graphene oxide powder was irradiated with 1 kW inside argon (Ar) plasma for 3 min at a pressure of 10 mbar in a quartz flask (1 L volume) connected to a vacuum pump and an argon mass flow meter (200 mL/min), followed by cooling to room temperature under Ar gas, washing the rGO samples with a solvent, and finally drying overnight at 80 °C. For the anchoring of MoS_2_ nanostructures, ∼140 mg of rGO nanosheets was dispersed in 35 mL of deionized water through ultrasonication for 1 h. Subsequently, 540 mg of ammonium molybdate tetrahydrate ((NH_4_)_6_Mo_7_O_24_·4H_4_O, 99.99% Sigma) and 1.14 g of thiourea (CH_4_N_2_S, 99.8%, Sigma), as molybdenum (Mo) and sulfur (S) precursors,^29^ were stirred into the rGO dispersion for 45 min, following which the dispersion was transferred to a 100 mL Teflon-lined autoclave and then heated at 220 °C for 24 h (Figure S1). Likewise, the free-standing MoS_2_ nanostructures were synthesized by using the same hydrothermal procedure. After cooling to room temperature, the black precipitate was thoroughly washed with ethanol and distilled water via centrifugation and then dried overnight at 70 °C.

### 2.2 Characterization of Active Materials

The structural disorders were investigated using a Renishaw inVia Rama Microscope equipped with a laser excitation wavelength of 532 nm. Average *I*_D_/*I*_G_ ratios were calculated from five measurements per sample. Surface functionalities of the samples were determined using a Nicolet iS50R FTIR spectrometer with a universal attenuated total reflectance (ATR) accessory. The morphological features of the samples were ascertained by using a Tescan Lyra dual beam scanning electron microscope (SEM) and the JEOLJ2010 transmission electron microscope (TEM). The thickness of the pristine MoS_2_ and the 2*D*/2D MoS_2_@rGO flakes was determined using atomic force microscopy (AFM) on an Ntegra Spectra microscope (NT-MDT). Following the dispersion of the samples in DMF for 30 min via ultrasonication, the sample was drop-cast on a freshly cleaved mica substrate, and surface scans were performed in the tapping mode under ambient conditions. The measurements were acquired with a scan rate of 1 Hz and a scan line of 512 using cantilevers with a strain constant of 1.5 kN m^–1^ equipped with a standard silicon tip with a curvature radius lower than 10 nm. The surface area was determined from the N_2_ adsorption and desorption isotherms by using a Micromeritics Tristar 3000 system. The specific surface area and the porosity were characterized using the Brunauer–Emmett–Teller (BET) method through adsorption–desorption measurements of N_2_ and the pore distribution analysis at the freezing temperature of N_2_ (77K). The crystallinity of the samples was analyzed using a Bruker D8 Advanced X-ray diffractometer (XRD, Bruker, Billerica, MASS, USA), equipped with a Cu-Kα radiation source. Finally, the surface chemical compositions of the samples were recorded on the ESCAProbeP X-ray photoelectron spectrometer (XPS) equipped with a monochromatic Al-α radiation source. Extended X-ray absorption fine structure (EXAFS) data were collected on the B18 beamline at the Diamond Light Source (DLS)^30^ at the Mo K-edge. Data were acquired in transmission and fluorescence modes with a Si311 monochromator and Pt-coated mirrors. The pure MoS_2_ and MoS_2_@rGO samples before the NRR were ground into a fine powder using a pestle and mortar and mixed with cellulose before being pressed into pellets and mounted on the sample holder. The exact masses of the samples used to create pellets of MoS_2_ and MoS_2_@rGO before the NRR were 3.89 and 9.46 mg, respectively. Cellulose was added to reach a total pellet mass of ca.. 60 mg. The MoS_2_ and MoS_2_@rGO after the NRR, given the nature and relatively low amount of samples available, were measured as foils sealed between two pieces of Kapton tape and mounted on a sample holder. EXAFS data were acquired in the 19800–21000 eV range corresponding to a *k-*range of up to 16 Å^–1^ with an energy resolution of 0.3 eV. EXAFS data analyses were performed with Athena software from the Demeter package.^31^

### 2.3 Electrochemical Measurements

The electrochemical performance of the as-prepared nanocatalysts was recorded at room temperature on an Autolab PGSTAT 204 (Metrohm, Switzerland) potentiostat using a gastight H-type electrolytic three-electrode system (Figure S2). For both electrochemical (cyclic voltammetry (CV) and linear sweep voltammetry (LSV) at 100 mV s^–1^ scan rate) and NRR measurements, MoS_2_@rGO and/or MoS_2_ nanocatalysts immobilized on the glassy carbon L-electrode (GCE, 5 mm in diameter) functioned as the working electrode, while the platinum (Pt) sheet and the Ag/AgCl electrode were the counter and reference electrodes, respectively, and 0.1 M KOH aqueous solution was the electrolyte. The modified GCE and the Ag/AgCl electrode, as the cathodic component, were separated from the counter electrode, the anodic component, by the pretreated Nafion 211 membrane.^32^ Prior to the measurements, the electrocatalyst ink was prepared by dispersing 4 mg of the nanocatalyst in 970 mL of absolute ethanol (EtOH, 99.8%, Merk) containing a 17 μL Nafion ionomer solution (5 wt %, Aldrich) by ultrasonicating for 1 h.^29^ Afterward, 10 μL of catalyst ink was drop-cast on the 0.1 mm Al_2_O_3_ slurry precleaned GCE surface and dried under ambient conditions to form the working electrode. Finally, the cathodic and anodic components were continuously purged with high-purity N_2_ (99.999%) at a constant flow rate of 20 mL min ^–1^, from which the ammonia (NH_3_) and the byproduct hydrazine (N_2_H_4_), produced from NRR, were collected in the 30 mL acid trap (0.1 M H_2_SO_4_). All potentials were referenced versus Ag/AgCl unless mentioned otherwise.

### 2.4 Quantification of Ammonia and Hydrazine

The amounts of the produced NH_3_ and its N_2_H_4_ byproduct from the NRR process were determined using the HI83300 multiparameter photometer (Hanna Instruments, Rhode Island). Using the ASTM D1426 Nessler method^33,34^ for determination of the concentration of NH_3_, 1 mL of the unreacted sample was pipetted into the 10 mL cuvette, following which 9 mL of ammonia high-range reagent B was used to bring the cuvette contents to the mark. After obtaining the zero background, four drops of ammonia high-range reagent A were added to the cuvette and mixed thoroughly, and after awaiting the color development, absorbance of the sample was measured at 420 nm. The yield rate of ammonia (Y.R._NH3_) was estimated according to eq 1
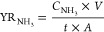
1where *C*_NH_3__ is the concentration reading from the photometer in mg·L^–1^*, V* is the volume of the acid trap in mL, *t* is the reduction time in seconds, and *A* is the area of the GCE in cm^2^. Ultimately, the faradaic efficiency (FE) of the NRR process was calculated based on eq 2
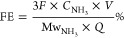
2where *C*_NH_3__ and *V* are described above, *F* is the faradaic constant, Mw_NH_3__ is the molecular weight of ammonia (17 g mol^–1^), and *Q* is the accumulated charge of the electrode during the NRR process.

For quantification of the hydrazine concentration after the NRR process, the D1385 *p*-dimethylamino-benzaldehyde method was used. Typically, two 10 mL cuvettes were filled with the unreacted sample and deionized water, respectively. To each cuvette, 12 drops of the hydrazine reagent were added, after which the cuvette containing the deionized water was used for zero background correction, and the sample containing the hydrazine reagent was used for measurement of absorbance at 466 nm. Similar equations were used to determine the amount of the byproduct N_2_H_4_, whereby *C*_N_2_H_4__ was the concentration reading from the photometer in μg·L^–1^ and Mw_N_2_H_4__ was the molecular weight of hydrazine (32 g mol^–1^).

## 3 Results and Discussion

### 3.1 Morphology and Adsorption Properties

**Figure 1:**
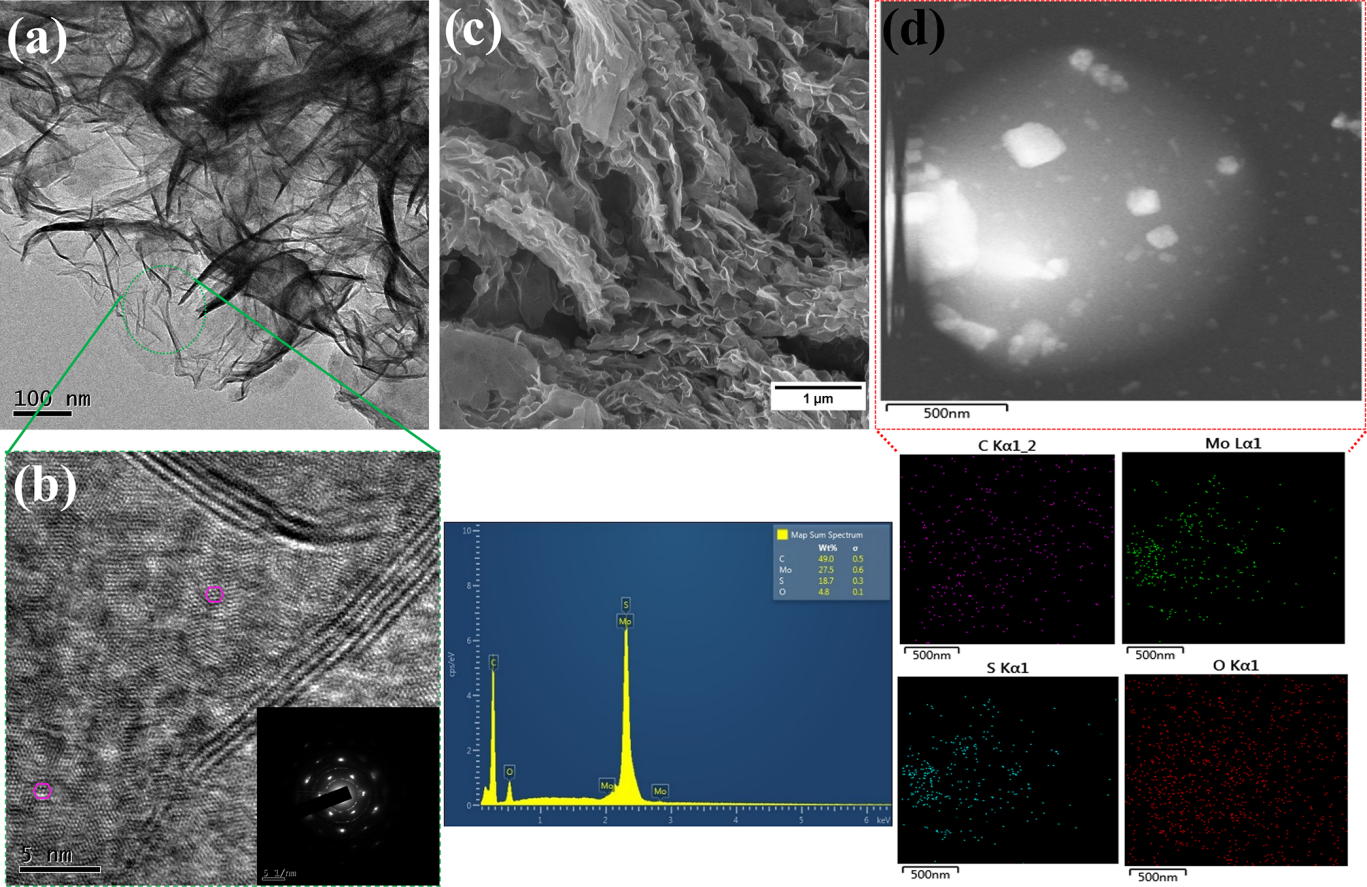
(a) Low-magnification, (b) high-resolution TEM, (c) SEM micrographs, and (d) TEM elemental mapping profiles of the MoS_2_@rGO hybrid samples before the NRR experiments.

The morphology of the composite nanocatalysts was investigated by using scanning electron microscopy (SEM), high-resolution transmission electron microscopy (HRTEM), and atomic force microscopy (AFM) characterization techniques. The HRTEM images showed thin nanosheets with a well-defined honeycomb atomic arrangement of MoS_2_ embedded within the semicrystalline sea of rGO (Figure 1a), thereby confirming the crystallinity of the MoS_2_ samples, as later highlighted by the XRD patterns. The SEM micrographs of the MoS_2_@rGO samples showed irregular nanosheets of MoS_2_ decorating the wrinkled sheets of rGO, both at the edges and in between the rGO nanosheets, somehow depicting a 3D-like nanostructure (Figure 1b). The observed 3D-like morphology could be essential for maximizing accessibility to active sites during the NRR electrocatalytic reactions, essentially leading to improved catalytic activity. On the other hand, the pristine MoS_2_ nanostructures revealed an agglomeration of well-defined MoS_2_ nanosheets into flower-like nanoclusters (Figure S3a). The absence of the nanoclusters on the MoS_2_@rGO hybrid samples is suggestive of the abundance of catalytically active sites on the rGO nanosheets, thus providing sufficient anchoring sites for the attachment and growth of MoS_2_ nanosheets. Thickness estimation of the MoS_2_ and MoS_2_/rGO flakes was conducted via AFM. As shown in figure S4c, the pure MoS_2_ flakes have an average thickness between 10 and 15 nm, whereas upon coupling with rGO, the thickness increases to 25–30 nm (Figure S4a). Interestingly, the heterojunction nanostructures tend to form agglomerated clusters (Figure S4b), which could be ascribed to the strong interface interactions between the two 2D structures.

Given the 3D-like network of the MoS_2_@rGO nanocatalysts, a type IV N_2_ adsorption/desorption isotherm with an H3 hysteresis loop (Figure S5) was evident at a relative pressure *P*_S_/*P*_0_ of 0.5–0.9.^35^ The observed isotherms corresponded to the formation of nonporous materials containing an assemblage of narrow slit-like pores due to the planar morphology of MoS_2_ and rGO nanosheets.^36^ Ultimately, the BET specific surface area (SSA) of MoS_2_@rGO was determined to be 56.1 m^2^ g^–1^ (Table S1), which was slightly larger than that of the MoS_2_ nanoclusters (45.12 m^2^ g^–1^). The high SSA for MoS_2_@rGO hybrids could subsequently be viewed as a crucial parameter for providing more catalytically active sites for NRR, in so doing facilitating the rapid production of ammonia.

### 3.2 Structure of Hybrid Nanomaterials

**Figure 2:**
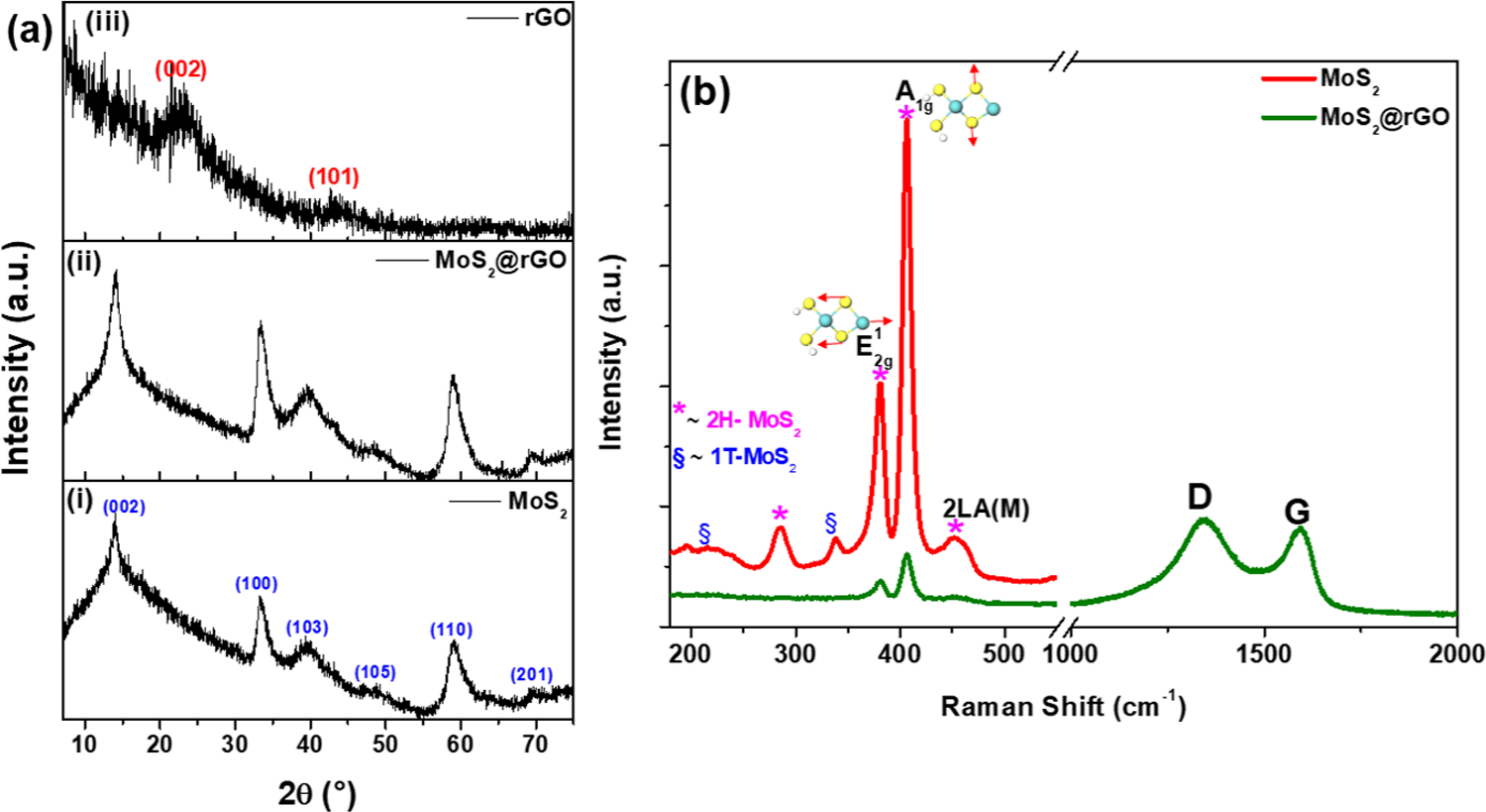
(a) XRD patterns and (b) Raman spectra of the pristine MoS_2_ and MoS_2_@rGO hybrid samples.

The crystallinity of the MoS_2_ and MoS_2_@rGO nanocatalysts was determined from the X-ray diffractograms between 10 and 80° (Figure 2a). The peaks at ∼14, ∼33, ∼38.9, ∼48, ∼59, and ∼70° for the diffraction patterns of both MoS_2_ and MoS_2_@rGO samples (Figures 2a (i) and (ii)) were indexed to the (002), (100), (103), (105), (110), and (201) reflections of the hexagonal 2H-MoS_2_, respectively (JCPDS no. 37–1492).^37^ The strong (002) diffraction plane (Figure 2a (ii)) for the MoS_2_@rGO sample, attributed to scattering of Mo–Mo between layers, indicated a better crystallinity of MoS_2_ nanosheets. Its slightly broader half-width could be attributed to the lattice defects, which could effectively increase the catalytic active sites.^38^ For comparison, the XRD pattern of the pristine rGO was also recorded, as shown in Figure 2a (iii). The broad diffraction peaks at ∼23 and ∼41° correspond to the (002) and (101) lattice planes of disordered carbon, respectively.^39^ However, the absence of the diffraction planes of rGO for the MoS_2_@rGO sample can be attributed to the low content of the carbonaceous component in the hybrid nanocatalysts. More importantly, the distinction between the types of MoS_2_ in both samples, viz. octahedral 1T-MoS_2_ and hexagonal 2H-MoS_2_, was unclear given that there was no sign of diffraction patterns associated with 1T-MoS_2_ due to the fact that most XRD peaks of 1T-MoS_2_ overlap with those of 2H-MoS_2._^37^ As such, the samples were further analyzed using Raman spectroscopy.

In order to determine the nature of the MoS_2_ anchored on the reduced graphene oxide nanostructures, Raman spectroscopy measurements were carried out as shown in Figure 2b. The spectra of the MoS_2_@rGO nanocatalysts displayed two characteristic first-order Raman bands corresponding to the E_2g_^1^ (∼381.4 cm^–1^) and A_1g_ (∼406.1 cm^–1^) vibration modes within the S–Mo–S layer of 2H-MoS_2_^40,41^ Likewise, formation of the 2H-MoS_2_ phase in the pristine MoS_2_ sample was shown by the presence of first-order Raman peaks located around ∼284.5, ∼380.6, and ∼406.4 cm^–1^, corresponding to the E_1g_, E_2g_^1^, and A_1g_ vibration modes.^40−43^ In addition to the 2H-MoS_2_, two minor vibrational modes at ∼215.7 and ∼337.9 cm^–1^ corresponded to the phonon modes in 1T-MoS_2_, thus indicating that the free-standing MoS_2_ nanoclusters comprise a mixture of octahedral and planar nanostructures.^40,41^ On the contrary, due to the strong interface interactions as well as structural compatibility between MoS_2_ and rGO,^20,23,25^ the results showed that predominantly hexagonal MoS_2_ nanosheets are effectively anchored on the large area surface of rGO.

In addition to the MoS_2_ vibrational modes, the Raman spectra of the MoS_2_@rGO nanocatalysts also exhibited two first-order Raman peaks for the carbonaceous materials, located at ∼1346 cm^–1^ (D band) and ∼1588 cm^–1^ (G band), indicating the presence of reduced graphene oxide in the composite nanocatalysts.^44,45^ The defect-level indicator was determined from the estimated ratio of the integrated area under the D band to that of the G band (vis. *I*_D_/*I*_G_). Given the relatively high defect density ratio (∼3.17), a better NRR performance can be anticipated from these unique defect-rich MoS_2_@rGO nanocatalysts due to the combined contribution from the few-layered MoS_2_ nanosheets, the highly defective rGO supports, and the synergetic effect of the two 2D structures. The surface functionalities on the MoS_2_@rGO samples were determined from Fourier transform infrared spectroscopy (FTIR, Figure S6). It is noteworthy to highlight that the absorption region between ∼1900 and 2100 cm^–1^ is associated with the modes from the diamond crystal on the instrument. Lack of a strong and broad absorption band at ∼3300–3500 cm^–1^, arising from the stretching vibrations of the OH bonds of the hydroxyl and carbonyl groups, confirmed the successful reduction of GO into rGO upon microwave irradiation as well as during the hydrothermal anchoring process.^46^ Additionally, the MoS_2_@rGO nanocatalyst spectrum exhibited peaks at ∼1303, ∼1630, and ∼2600 cm^–1^, corresponding to the C–OH carbonyl, C=C in-plane, and C–H alkyl stretching bands in rGO.^46^ Furthermore, a minor peak at ∼10 66 cm^–1^ represented the S–OH asymmetrical stretching modes, suggesting minor surface functionalization of rGO with sulfophenyl groups during the hydrothermal process.^47^ For the pristine MoS_2_ sample, the vibrational bands at ∼1066, ∼961, ∼835, and ∼631 cm^–1^ showed the formation of MoS_2_ nanostructures.^48,49^

### 3.3 Surface Composition Determination

**Figure 3:**
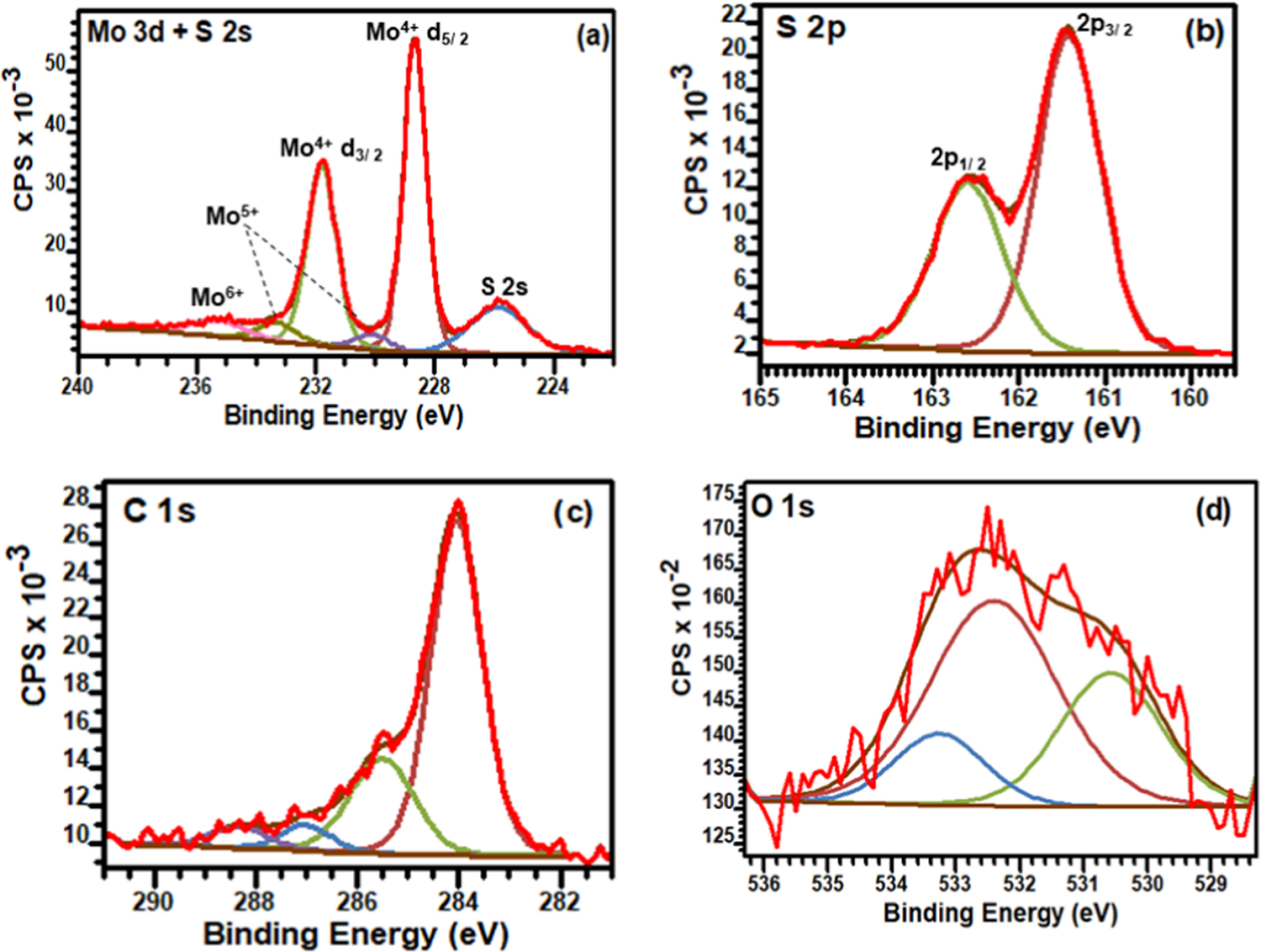
Deconvoluted (a) Mo 3d + S 2s, (b) S 2p, (c) C 1s, and (d) O 1s spectra of the MoS_2_@rGO sample.

The chemical valence states of the elements in the nanocatalysts as well as the surface compositions were determined by using X-ray photoelectron spectroscopy (XPS). The survey spectra (Figure S7) of the MoS_2_@rGO and MoS_2_ samples revealed the presence of Mo 3d (∼229 eV), S 2p (∼162 eV), C 1s (∼284 eV), N 1s (∼395 eV), and O 1s (∼532 eV). The S/Mo element ratio of the hybrid nanocatalysts was estimated from the integral peak area of the XPS survey spectra and was determined to be ∼2.0. The carbon and nitrogen peaks could be ascribed to the contribution of the precursor salts. Figure 3 displays the peak fitted XPS spectra for the MoS_2_@rGO sample, while those of the pristine MoS_2_ are shown in figure S7. The high-resolution Mo 3d spectrum (Figure 3a) consists of component peaks located at 228.6, 230.1, 231.8, 233.2, and 235.3 eV. The two main peaks at 228.6 and 231.8 eV are assigned to Mo^4+^ d_5/2_ and Mo^4+^ d_3/2_ of the 2H phase, while the component peak at a lower binding energy (225.8 eV) is attributed to S 2s.^42,50^ The presence of the 1T phase on the pristine MoS_2_ sample was shown by additional component peaks at 228.2 and 231.2 eV (Figure S7b), corresponding to the Mo^4+^ d_5/2_ and Mo^4+^ d_3/2_ oxidation states of the 1T phase.^42,50^ The binding energies at 229.1 and 232.3 eV corresponded to Mo^5+^ d_5/2_ and Mo^5+^ d_3/2_ regions.^50,51^ Compared to the pristine MoS_2_ sample (Figure S7), the binding energies of Mo^4+^ in the MoS_2_@rGO are slightly negatively shifted. The negative shift of the Mo^4+^ species can be ascribed to the interaction of the Mo atoms with the graphene lattice. The existence of the fifth XPS peak at 235.7 eV can be indexed to Mo^6+^ 3d_5/2_, indicating regions of surface oxidation of MoS_2_.^50,52^

Last but not least, the core-level spectrum of the S 2p peak (Figure 3b) was resolved into a doublet corresponding to S 2p_3/2_ (∼161.4 eV) and S 2p_1/2_ (∼163.9 eV), respectively. This represents the presence of bridging S_2_^2–^-type species bonded to Mo^4+^ oxidation states.^50,52^ Ambiguously, the component peak at ∼163.9 eV can be assigned to C–S–C bonding and indicates the introduction of S atoms into the defective graphene lattice.^53,54^ The C 1s spectrum for the MoS_2_@rGO sample (Figure 3c), was fit with four components centered at 284.1, 285.4, 286.8, and 288.3 eV corresponding to the presence of graphitic sp^2^ C=C bonds, defect-induced C–C bonds, and the oxygenated carbon bonds, respectively.^55,56^ Finally, the O 1s spectra (Figures 3d and S7d) showed peaks at 533.3, 532.5, 531.7, and 530.8 eV, representing the contribution from Mo–O and/or C–OH, C–O, and O=C bonding configurations.^50^

### 3.4 Electrochemical Performance

**Figure 4:**
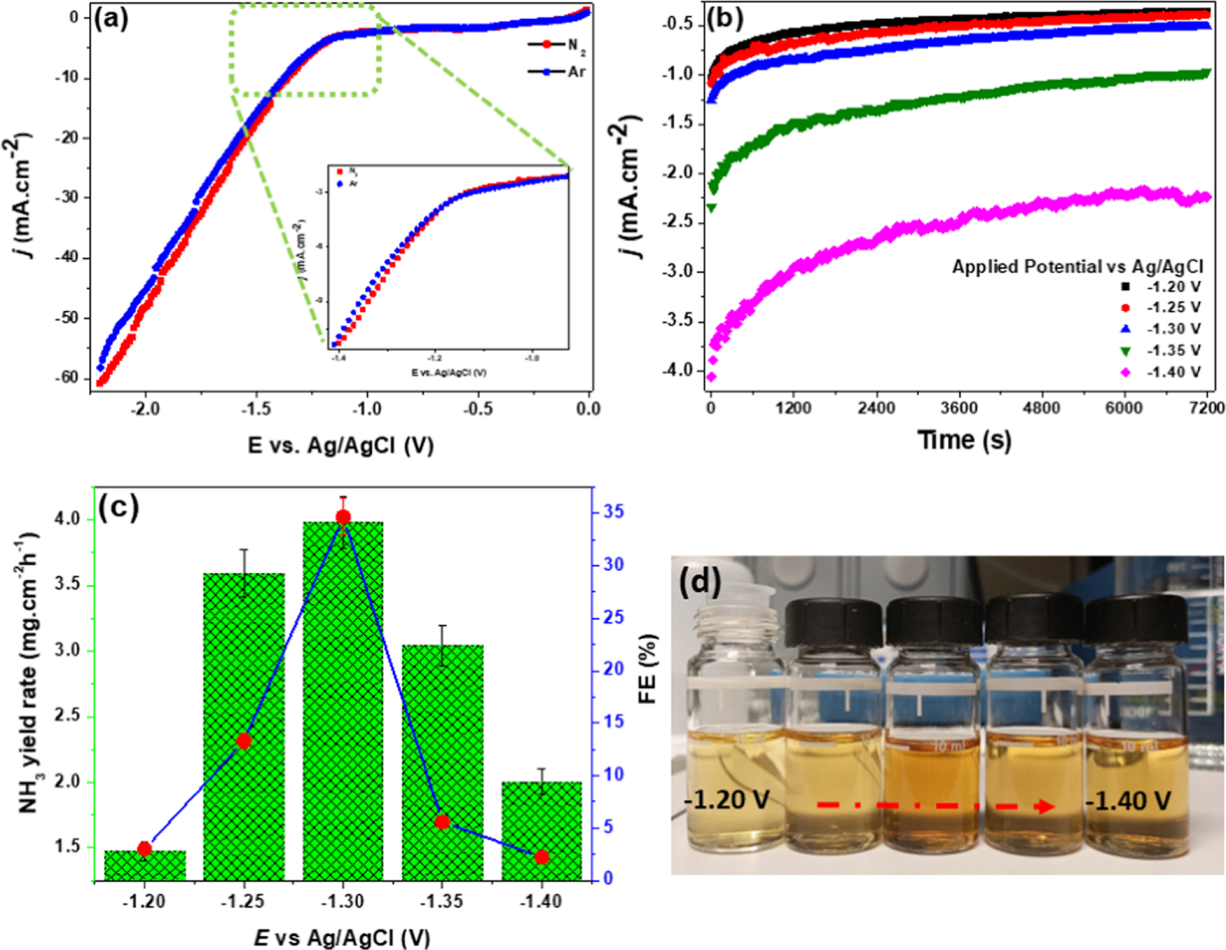
(a) Polarization curves of MoS_2_@rGO nanocatalysts in N_2_- and Ar-saturated 0.1 M KOH electrolyte. (b) Chronoamperometric (*j–t*) curves at various potentials. (c) NRR performance of MoS_2_@rGO nanocatalysts (NH_3_ yield rate and FE) versus cathodic potential. (d) Corresponding color solutions at −1.20 V (left) to −1.40 V (right) after NRR experiments catalyzed by MoS_2_@rGO nanocatalysts.

Electrochemical methods were performed to verify the conjecture for the NRR performance of the MoS_2_@rGO electrocatalyst within a potential window of −2 to 0 V. Verification of the voltammetric NRR activity was performed by plotting the linear sweep voltammetric (LSV) polarization curves in 0.1 M KOH electrolyte solution saturated with N_2_ or Ar, as shown in Figure 4a. The reaction in argon-saturated electrolyte was performed so as to recognize the possible contributions to nitrogen production from ubiquitous N-containing contaminants such as adventitious NH_3_ in N_2_ gas supply, the residues from the Mo-precursor salt, or the electrolyte. A slightly higher cathodic current density (Figure 4a, inset) is evident within the sweeping potential range of −1.20 to 1.40 V during purging with N_2_ as compared to Ar purging. From the determination of the NRR performance after 2 h reaction time in an alkaline medium, the contribution to N_2_ production from potential contaminants could be considered to be practically minimal, as indicated by the NH_3_ yield rate of 0.054 mg h^–1^ cm^–2^ and 0.06% FE under Ar gas purging. To explore the reaction kinetics of the NRR process catalyzed by the MoS_2_@rGO electrocatalysts, the polarization curves as a function of reaction time (*j–t)* at different cathodic potentials were plotted as shown in Figure 4b. From the chronoamperometric experiments at different cathodic potentials (Figure 4c), a low NRR sensitivity (FE) of 2.98% at an NH_3_ yield rate of 1.48 ± 0.07 mg h^–1^ cm^–2^ was recorded for the −1.20 V sweeping cathodic potential. A gradual increase of the potential to −1.30 V saw a considerable NRR improvement (FE ≈ 34.7%) with a remarkable NH_3_ yield rate of 3.98 ± 0.19 mg h^–1^cm^–2^. However, an increase to more negative potentials (−1.40 V) showed a gradual loss of the NRR activity (FE ≈ 2.24%) and a consequently decreasing NH_3_ yield rate (2.0 ± 0.1 mg h^–1^ cm^–2^), thereby indicating potential increased dominance of the competitive HER process. Evidence of varying amounts of ammonia production at different cathodic potentials was revealed by the varying color intensities of the solutions (Figure 4d) after the Nessler method. The obtained NRR activity results showed the importance of the formation of hybrid structures for enhancing the NRR performance catalyzed by the MoS_2_@rGO nanocatalysts in comparison to the NRR process catalyzed by pristine MoS_2_ nanocatalysts (Figure S3a). In particular, MoS_2_ nanocatalysts led to a very low NRR sensitivity (FE) of 0.23% at an NH_3_ yield rate of 26.8 ± 0.07 μg h^–1^ per square area of the covered GCE surface at the overpotential of −1.20 V. Besides the recorded low NRR sensitivity on the MoS_2_ nanocatalysts (Figure S8a), due to the increased dominance of the competitive HER process within the sweeping potential window, the results also revealed poor selectivity of the MoS_2_ nanocatalysts for ammonia production to that of the hydrazine byproduct in comparison to the MoS_2_@rGO nanocatalysts (Figure S8b). In comparison with the reported hybrid catalysts of MoS_2_ and graphene derivatives or 2D structures in either acidic or neutral media (Table 1), the results revealed that the MoS_2_@rGO heterojunction nanocatalysts exhibited better electrocatalysis for the NRR in the alkaline media.

**Table 1:** Comparison of Electrolytic N_2_ Reduction Performance for MoS_2_-Based Composite Electrocatalysts under Ambient Conditions

catalyst^a^	electrolyte	NH_3_ yield (μg h^–1^mg_cat._^–1^)	FE %	potential (V vs RHE)	ref
MoS_2_-rGO/CP	0.1 M LiClO_4_	24.82	4.58	–0.45	(23)
MoS_2_ NDs-RGO/CC	0.1 M Na_2_SO_4_	16.41	27.93	–0.75	(25)
MoS_2_-_g_C_3_N_4_/CC	0.1 M LiClO_4_	18.5	17.8	–0.3	(22)
FeS_2_/MoS_2_@rGO/CC	M KOH	10.35	9.62	–0.4	(60)
FeS_2_/MoS_2_@rGO/CC	1.0 M K_2_SO_4_	41.1	38.6	–0.2	
MoS_2_@rGO/CC	1.0 M K_2_SO_4_	27.1	25.5	–0.3	
MoS_2_@BCCF/CC	0.1 M Li_2_SO_4_	43.4	9.81	–0.2	(61)
1T-MoS_2_@Ti_3_C_2_/GCE	0.1 M HCl	30.33	10.94	–0.3	(62)
1T’-MoS_2_@Ti_3_C_2_/GCE	0.1 M Na_2_SO_4_	31.93	30.75	–0.95	(63)
2H-MoS_2_@Ti_3_C_2_/GCE	0.1 M Na_2_SO_4_	26.41	5.23		
FeS_2_/MoS_2_@IF_x_/CC	0.1 M KOH	7.1 × 10^–10^^b^	4.6	–0.5	(64)
MoS_2_@rGO/GCE	0.1 M KOH	195.4	34.65	–0.3	this work

a MoS_2_, molybdenum disulfide; CP, carbon paper; NDs, nanodots; CC, carbon cloth; GCE, glassy carbon electrode; rGO, reduced graphene oxide; _g_C_3_N_4_, graphitic carbon nitride; FeS_2_, iron sulfide; BCCF, bacterial cellulose converted carbon fiber; Ti_3_C_2_, titanium carbide MXene; IF, iron framework.

b → mol s^–1^cm^–2^

**Figure 5:**
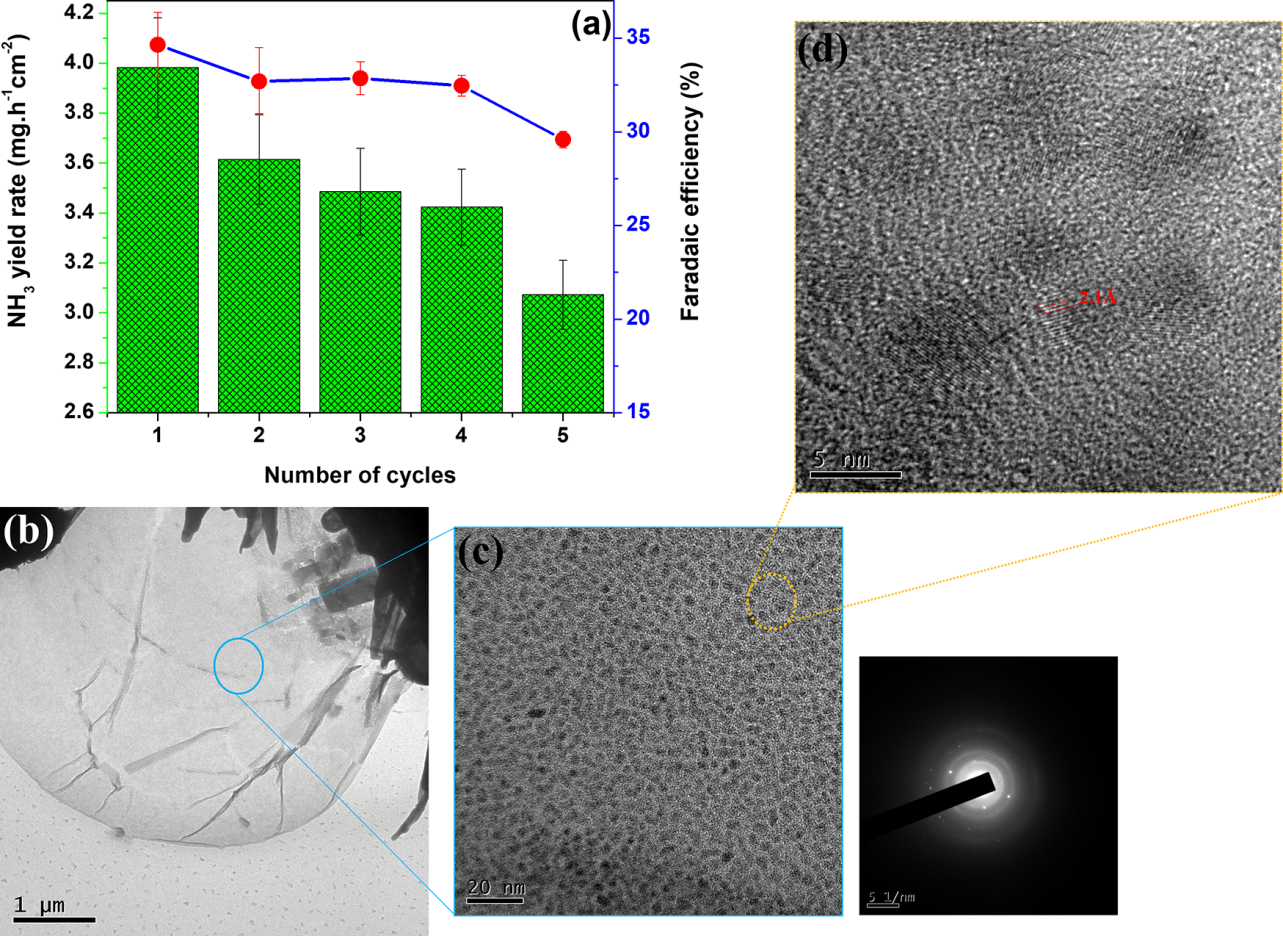
(a) Stability test of MoS_2_@rGO nanocatalysts (NH_3_ yield rate and FE) after five-cycle 2 h NRR experiments. (b–d) HRTEM micrographs after the first cycle of NRR experiment.

Durability studies constitute another key factor for evaluating a high-performing NRR catalyst for practical application, and this is typically measured by chronoamperometric analysis. As shown in Figure 5a, the MoS_2_@rGO nanocatalysts maintain a relatively stable NRR catalytic activity after multiple cycles of operation at −1.30 V. After ∼8 h of operation, 4 cycles with 2 h intervals, a steady-state production of NH_3_ was observed, retaining ∼88% of the sensitivity (FE). The outstanding durability could be attributed to the structural properties of the MoS_2_@rGO nanocatalysts, which subsequently guaranteed an enhanced NRR catalytic activity. For instance, regardless of the slight aggregation of crystalline MoS_2_ (size ∼2.8 ± 0.8 nm) within the rGO mass after the 2 h NRR experiments (Figure 5b–d), the preservation of the 2H phase of MoS_2_ assisted in retaining the nitrogen reduction activity. However, this aggregation could be presumed to be the cause for the drastic loss of activity with prolonged use of the MoS_2_@rGO nanocatalysts, as observed by only 20% FE after the fifth cycle of NRR experiments. Nonetheless, the thin morphology of the MoS_2_ nanosheets, despite their clustering, still provided a large surface area and a slightly mesoporous structure, thereby facilitating a rapid electron–ion transport pathway as well as abundant channels for the transport of reactant molecules. Moreover, the defect-rich rGO nanosheets provided sufficient active sites for the easy adsorption and dissociation of N_2_, whereas the interaction of Mo atoms with the graphene lattice enhanced the electrical conductivity (*R*_s_ ≈ 16.9 Ω), consequently ensuring faster electron transfer and low charge transfer resistance (*R*_ct1_ ≈ 74.3 Ω and *R*_ct2_ ≈ 30.4 Ω), as shown by the electrochemical impedance spectroscopy (EIS) Nyquist plots (Figure S9d and Table S4).

To ascertain the obtained NRR performance and sensitivity of the MoS_2_@rGO hybrid nanocatalysts in alkaline media, hydrogen evolution reaction (HER) activity of each electrocatalyst was established via plotting of the LSV curves in 1.0 M KOH electrolyte (Figure S9a). As observed in Figure 5c, MoS_2_@rGO nanocatalysts exhibit an HER Tafel slope of ∼214 mV dec^–1^, while the MoS_2_ nanocatalysts revealed a Tafel slope of ∼188 mV dec^–1^. The slow HER kinetics on the MoS_2_@rGO nanocatalysts could be attributed to the predominance of the 2H-MoS_2_ phase, thereby inhibiting the rate of HER due to the fact that the H^+^/H_2_ redox potentials lie above the conduction band energy level of the 2H-MoS_2_ phase,^41,57−59^ whereas in comparison to the pristine MoS_2_, the HER activity could primarily be slightly enhanced by the improved accessibility to the active sites. Based on these HER activity measurements, it is evident that engineering the form of MoS_2_ anchored on rGO nanosheets plays a pivotal role in promoting NRR, such that it becomes more competitive against HER.

### 3.5 N_2_ Adsorption and Reduction Mechanism

**Figure 6:**
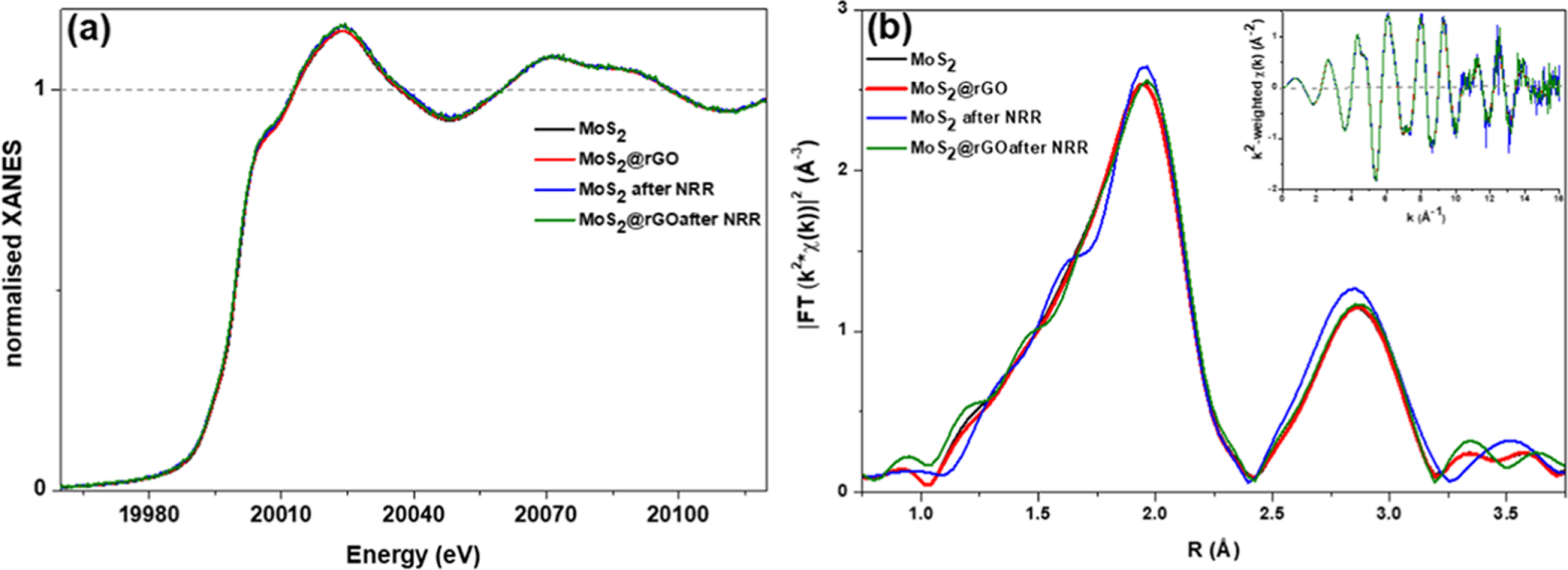
(a) Normalized XANES for MoS_2_ and MoS_2_@rGO samples before and after 3 h NRR measurements at the Mo K-edge. (b) EXAFS Fourier transform (FT) *k*^2^-weighted χ(*R*) signal for MoS_2_ and MoS_2_@rGO samples before and after NRR at the Mo K-edge. The *k-*range used for FT is 2.5–14.1 Å^–1^.

In order to shed light onto the structural and electronic differences between the samples that could lead to insights into the mechanism of the enhanced NRR activity observed for MoS_2_@rGO, we performed ex situ EXAFS and XANES measurements at the B18 beamline at the Diamond Light Source. The samples in question were MoS_2_ and MoS_2_@rGO before and after NRR. The four XANES spectra of the samples at the Mo K-edge, provided in Figure 6a of the Supporting Information, do not show any significant change before and after the reaction or in the presence (or absence) of the rGO substrate. This evidence suggests that the Mo p states do not contribute substantially to the reaction mechanism. Furthermore, in Figure 6b, we also report the Fourier-transformed EXAFS data, which show minimal variation in the intensities of the peaks. On this basis, we assert that there are minimal changes to the local structure and coordination geometry around the Mo atoms in all of the different samples. Notwithstanding the information acquired by XANES and EXAFS characterization, a detailed formulation of the mechanism behind the catalysis and associated changes upon interaction with the support remains an open question. It should be noted that X-ray absorption spectroscopy (XAS) captures an average over all of the Mo atoms in the beam; if a small fraction of Mo atoms is responsible for the catalytic activity or is modified by the interaction with the rGO support, these changes may not be detectable by XAS. Therefore, given all of the information, we can postulate that electrons in other Mo-orbitals (4d shell), or the presence of low-concentrated S-vacancies or rGO, are responsible for the enhanced NRR.

## 4 Conclusions

In summary, we have demonstrated the significance of catalyst design in achieving a highly active and selective NRR electrocatalyst through the formation of MoS_2_@rGO 2D/2D hybrid structures. The results indicated that the ultrathin layered structure, the relatively large surface area, and the defect-rich morphology of the MoS_2_@rGO 2D/2D hybrid electrocatalysts played an important role in the enhanced NRR performance, characterized by a high sensitivity (FE) of 34.7% with an NH_3_ yield rate of 3.98 ± 0.19 mg h^–1^ cm^–2^ at the overpotential of −0.3 V vs RHE in 0.1 M KOH solution. Moreover, the semiconducting behavior of the 2H-MoS_2_ phase further promoted the NRR process by providing additional active binding sites at S-vacancies while also inhibiting the rate of HER due to the difference in the energy levels between the H^+^/H_2_ redox potentials and the conduction band. By tuning the phases on MoS_2_ nanosheets, and thereby controlling their electrochemical properties, the study provided an inexpensive route for designing future 2D/2D hybrid electrocatalysts, which could also be useful for other energy conversion and storage systems that are typically limited by the HER process, such as carbon dioxide reduction reaction.

## Data Availability

Main data presented in this study are available at https://zenodo.org/records/,

## Supporting Information Available

Figure S1, synthesis procedure for anchoring MoS_2_ nanosheets on rGO nanosheets; Figure S2, representation of the H-type electrolytic cell; Figure S3, SEM, low-magnification TEM, and HRTEM micrographs of the pristine MoS_2_ samples, as well as SEM EDX mapping profiles; Figure S4, AFM images and height profiles for the MoS_2_@rGO hybrid, as well as pristine MoS_2_ samples; Figure S5, adsorption isotherms of pristine MoS_2_ and the MoS_2_@rGO hybrid samples; Figure S6, FTIR spectra of pristine MoS_2_ and the MoS_2_@rGO hybrid samples; Figure S7, XPS survey spectra of MoS_2_ and MoS_2_@rGO, as well as fitted Mo 3d + S 2s, S 2p, and O 1s spectra for MoS_2_ nanostructures; Figure S8, NRR performance of MoS_2_ nanocatalysts (NH_3_ yield rate and FE) versus cathodic potential, yield rate of hydrazine (N_2_H_4_) after NRR experiments catalyzed by MoS_2_ and MoS_2_@rGO nanocatalysts, polarization curves in N_2_ and Ar, as well as chronoamperometric curves at various potentials of MoS_2_ nanocatalysts in N_2_-saturated 0.1 M KOH electrolyte; Figure S9, polarization curves, overpotentials, and Tafel plots, as well as Nyquist plots of the electrocatalysts during HER activity in 1.0 M KOH; Table S1, textual properties of pristine MoS_2_ and the MoS_2_@rGO hybrid samples; Table S2, Raman parameters for the pristine MoS_2_ and the MoS_2_@rGO hybrid samples; Table S3, atomic compositions of the composite samples; and Table S4, EIS analysis of pristine MoS_2_ and the MoS_2_@rGO hybrid samples post HER experiments (PDF)
