# Supplementary material for: Enhancing Nitrogen Reduction Reaction through Formation of 2D/2D Hybrid Heterostructures of MoS2@rGO
Source: ACS Appl Mater Interfaces. 2024 Apr 30;16(19):24514–24. doi: 10.1021/acsami.4c00719 (PMC11103663; doi:10.1021/acsami.4c00719)
Supplement: Supplementary file 1 — am4c00719_si_001.pdf [file am4c00719_si_001.pdf]

Supporting Information

Joyce B. Matsoso; Email: matsosoj@vscht.cz

Zdeněk Sofer; Email: soferz@vscht.cz

Enhancing nitrogen reduction reaction through formation of 2D/2D hybrid heterostructures of MoS_2@rGO

Joyce B. Matsoso[1, 2]*, Nikolas Antonatos[1,3], Lukáš Dekanovský[1], Roussin Lontio Fomekong [1]†, Joshua Elliot[4], Diego Gianolio[4], Vlastimil Mazánek[1], Catherine Journet [2], Zdeněk Sofer[1]*

[1] Department of Inorganic Chemistry, University of Chemistry and Technology in Prague, Technická 5, 166 28 Prague 6, Czech Republic

[2] Laboratoire des Multimatériaux et Interfaces, UMR CNRS 5615, Univ-Lyon, Université Claude Bernard Lyon 1, F-69622 Villeurbanne Cedex-France

[3]Department of Semiconductor Materials Engineering, Faculty of Fundamental Problems of Technology, Wrocław University of Science and Technology, Wybrzeże Wyspiańskiego 27, 50-370 Wrocław, Poland

[4]Diamond Light Source, Diamond House, Harwell Science and Innovation Park, Didcot, Oxfordshire OX11 0DE, United Kingdom

Experimental:

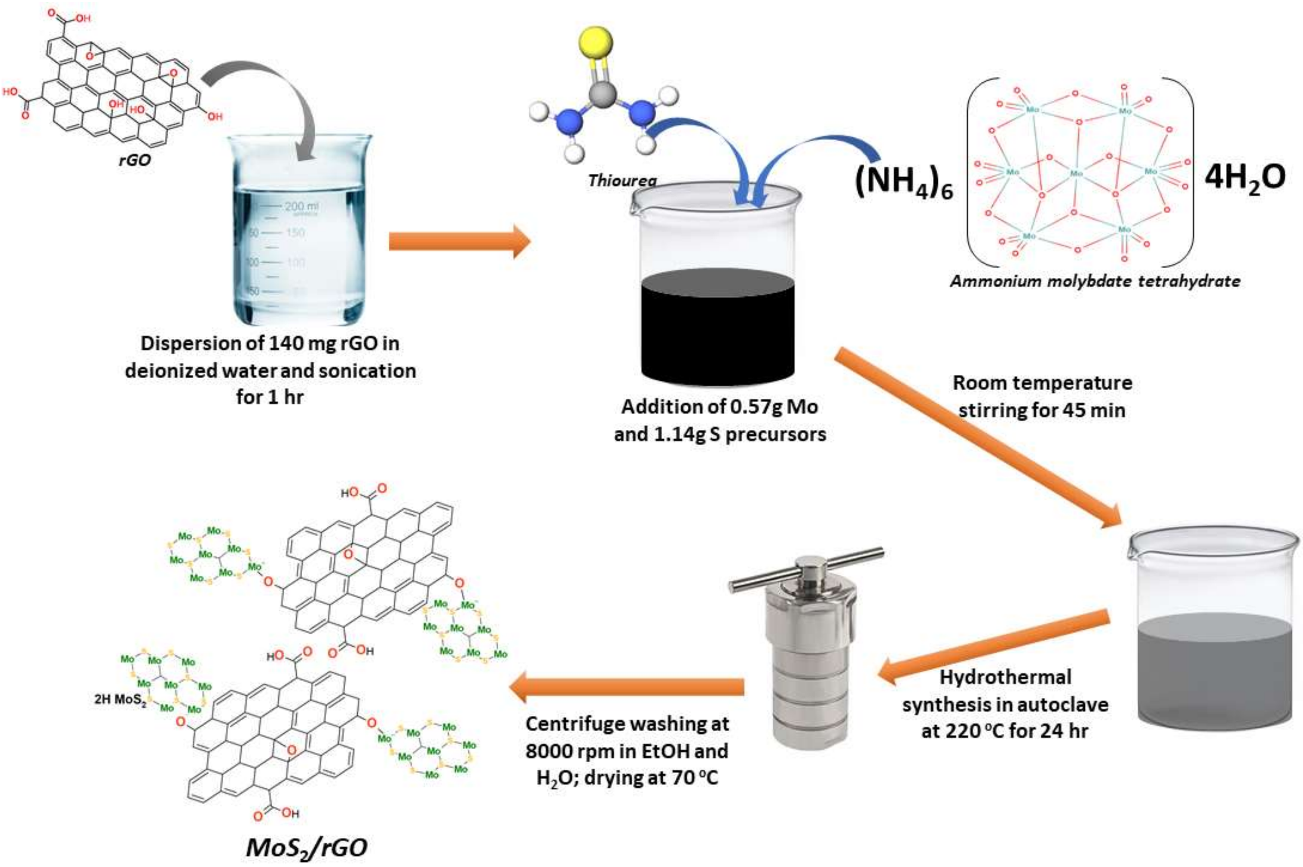

Figure S1: Synthesis procedure for anchoring MoS_2 nanosheets on rGO nanosheets.

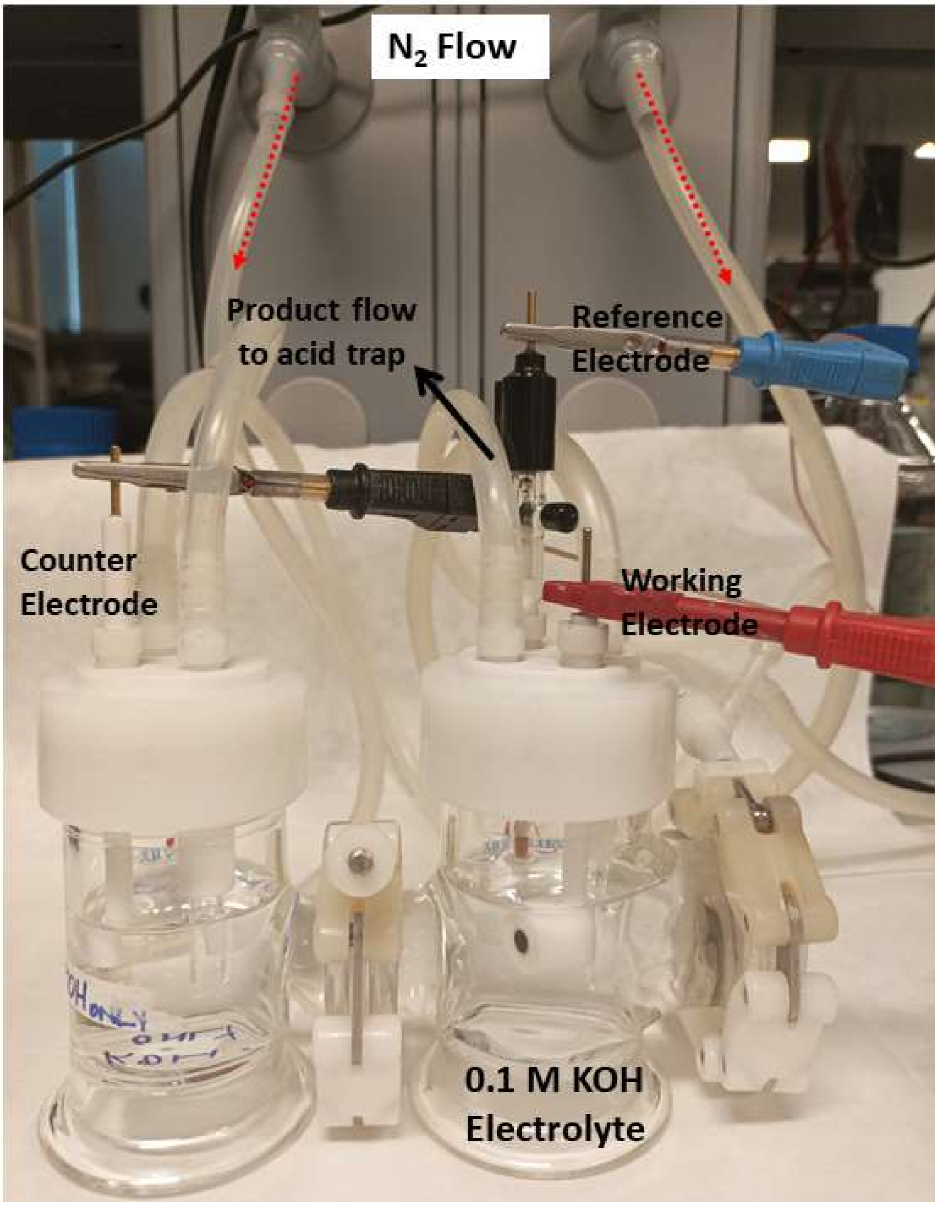

Figure S2: Representation of the H-type electrolytic cell.

Results:

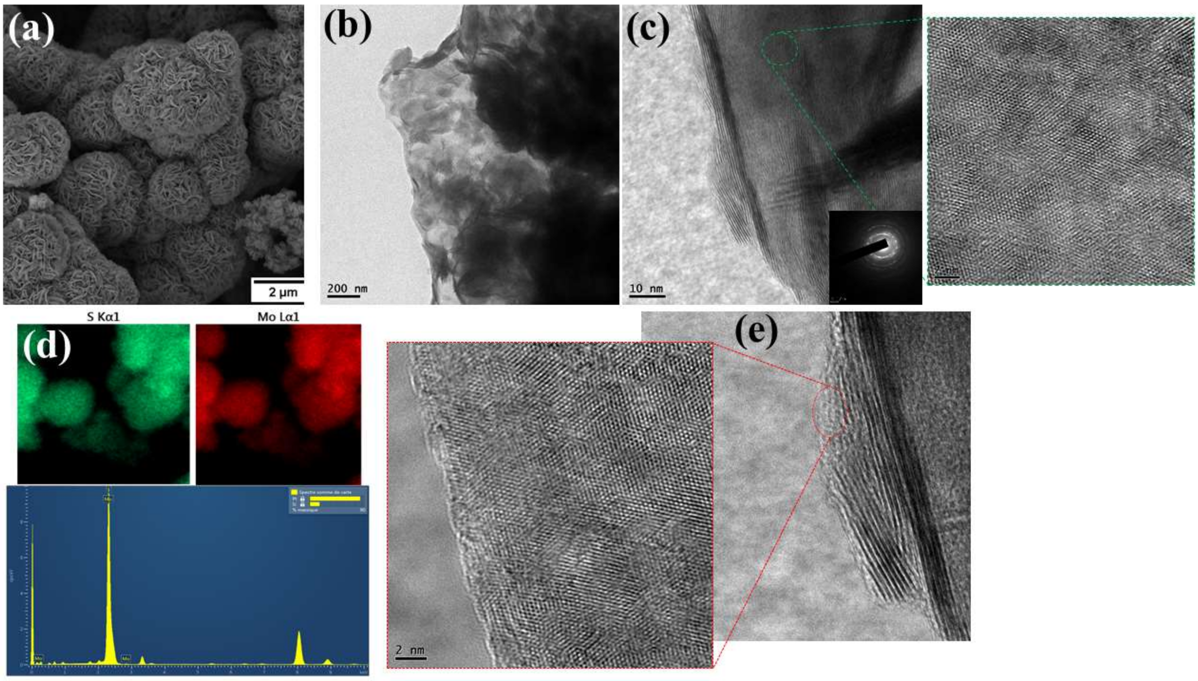

Figure S3: (a) SEM, (b-c, e) low magnification TEM and HRTEM micrographs of the pristine MoS_2 samples, as well as (d) SEM EDX mapping profiles.

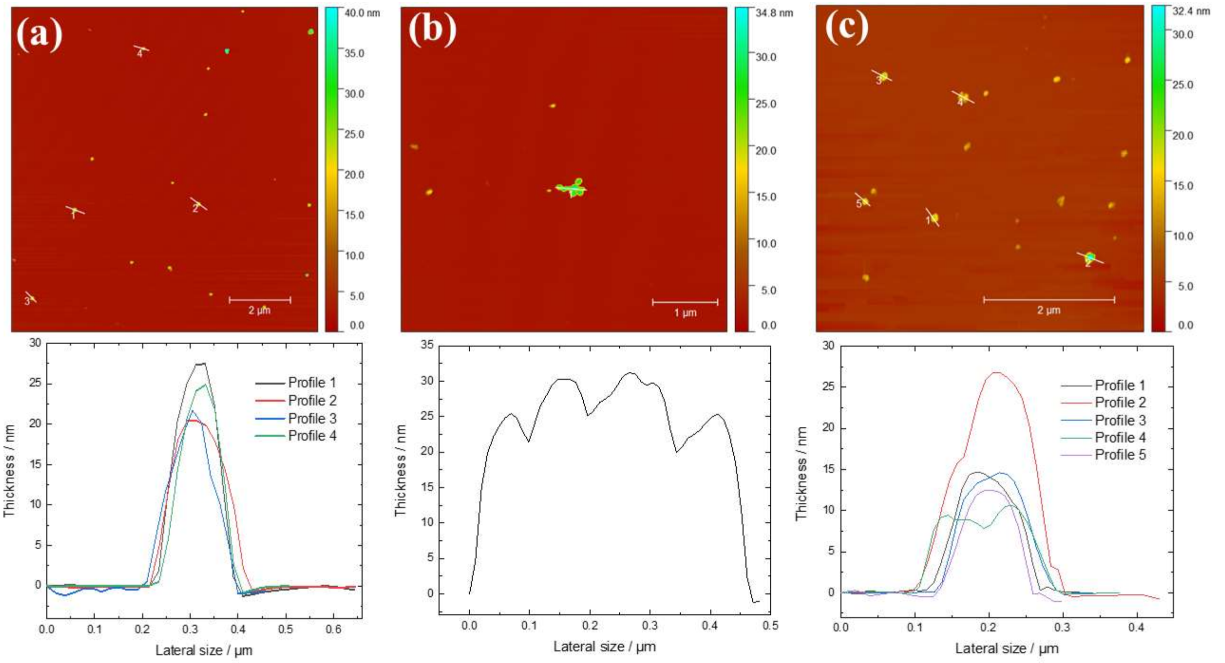

Figure S4: AFM images and height profiles for the (a) and (b) MoS_2@rGO hybrid, as well as (c) pristine MoS_2 samples.

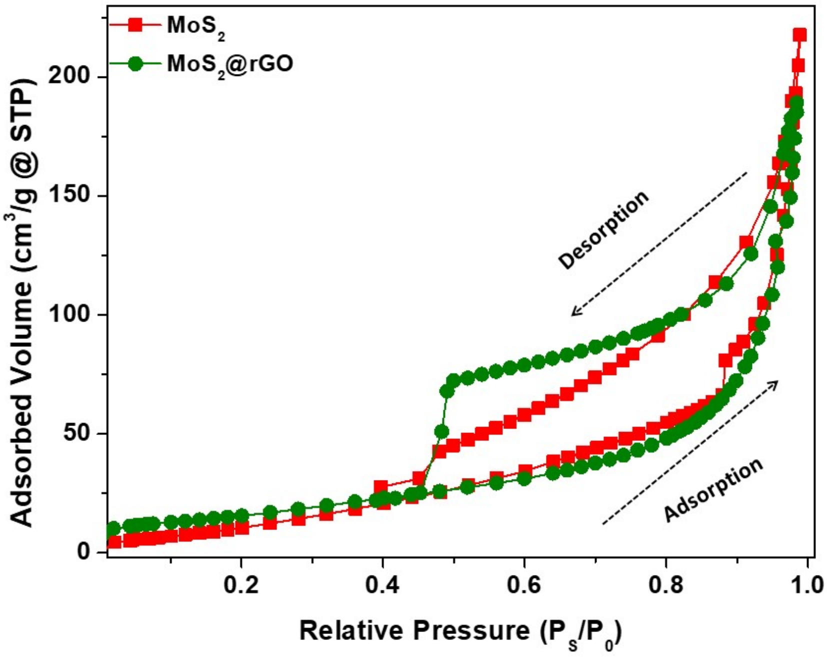

Figure S5: Adsorption isotherms of pristine MoS_2 and the MoS_2@rGO hybrid samples.

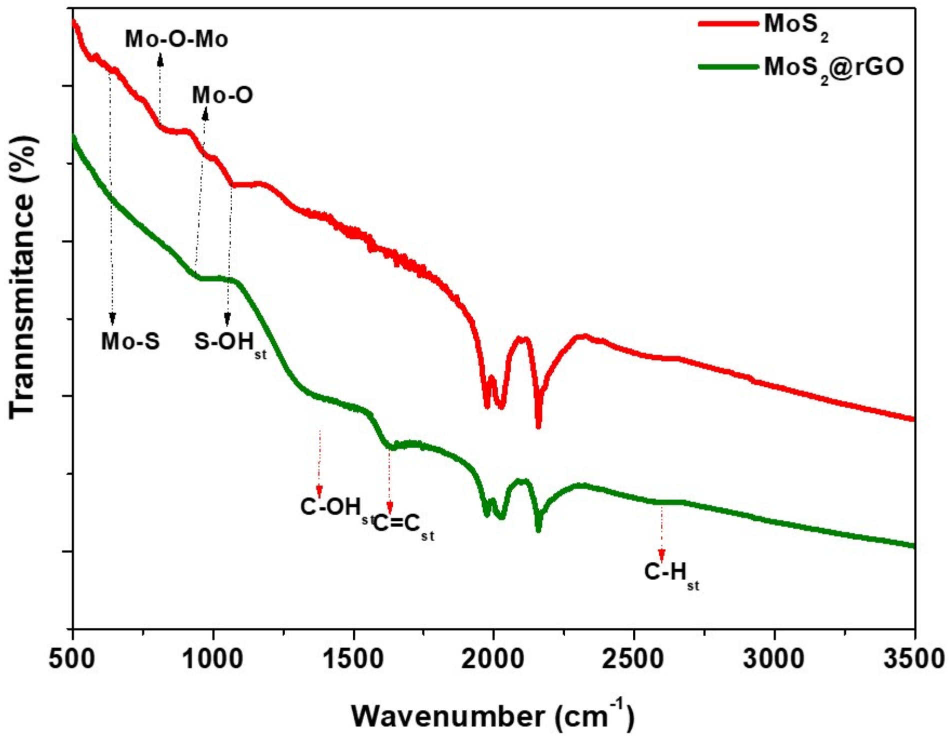

Figure S6: FT-IR spectra of pristine MoS_2 and the MoS_2@rGO hybrid samples.

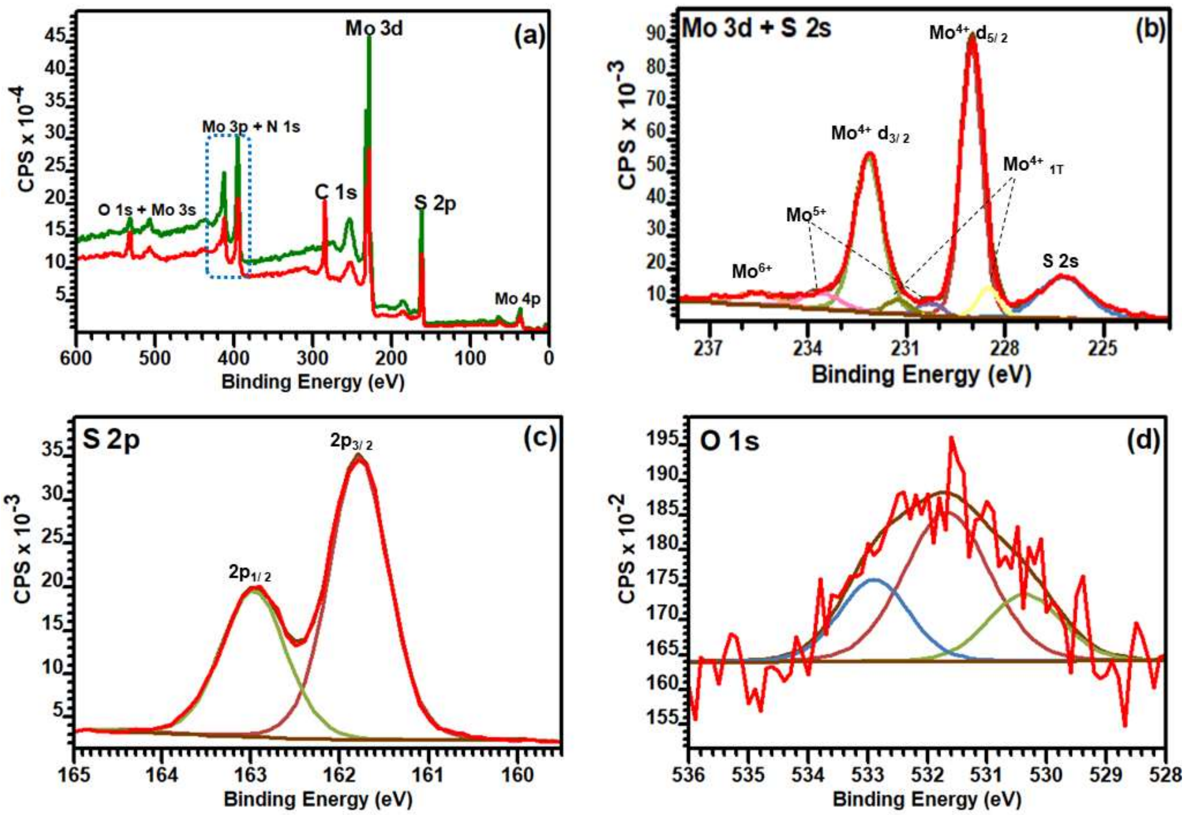

Figure S7: (a) XPS survey spectra of MoS_2 (***green***) and MoS_2@rGO (***red***), as well as fitted (b) Mo 3d + S 2s, (c) S 2p, and (d) O 1s spectra for MoS_2 nanostructures.

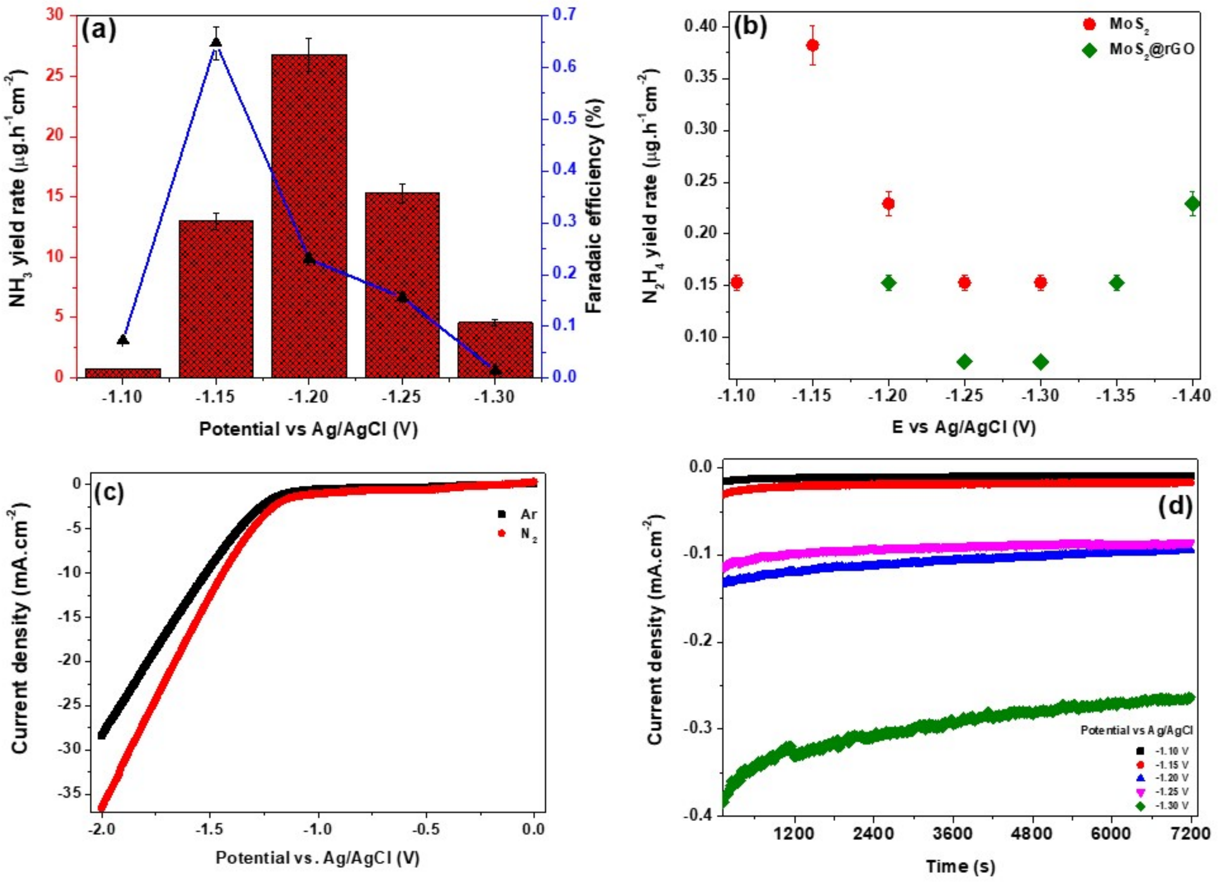

Figure S8: (a) NRR performance of MoS_2 nanocatalysts (NH_3 yield rate and FE) versus cathodic potential, (b) Yield rate of hydrazine (N_2H_4) after NRR experiments catalyzed by MoS_2 and MoS_2@rGO nanocatalysts, (c) Polarization curves in N_2 and Ar, as well as (d) Chronoamperometric (*j-t*) curves at various potentials of MoS_2 nanocatalysts in N_2 saturated 0.1 M KOH electrolyte.

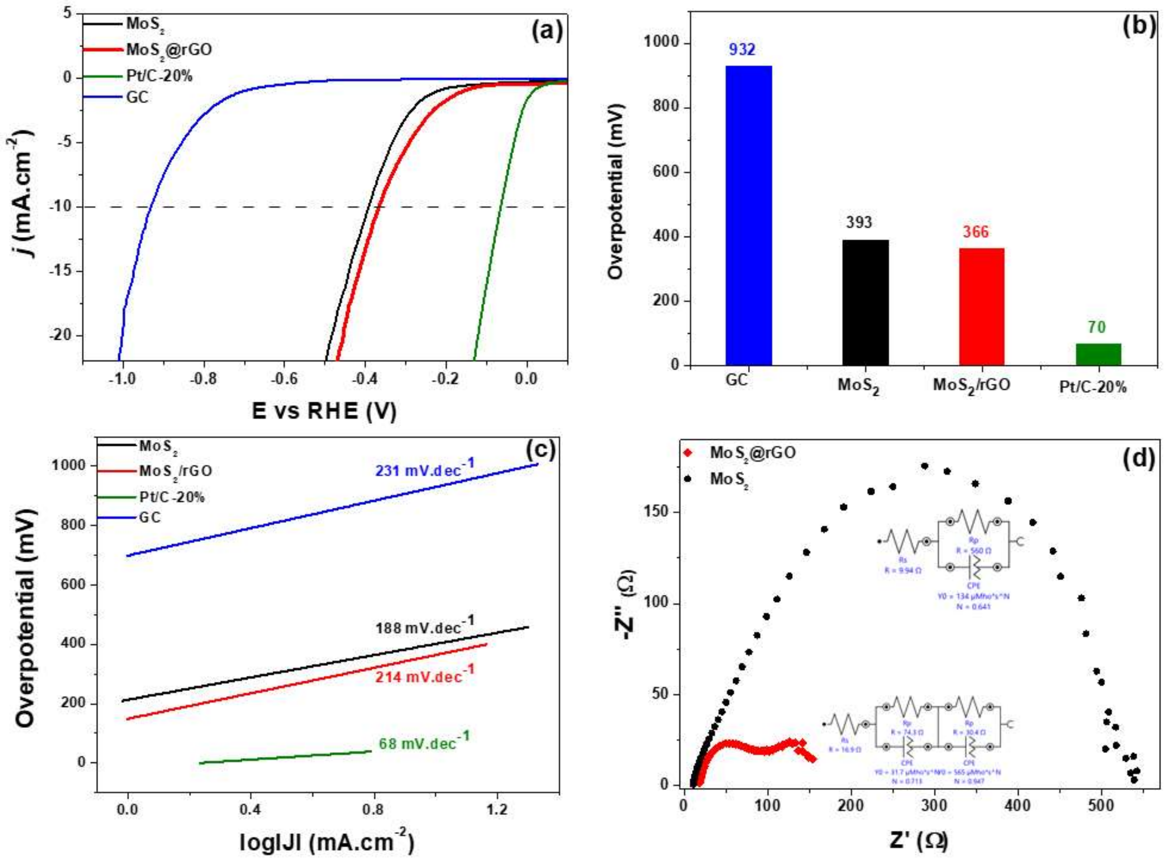

Figure S9: (a) Polarization curves, (b) Overpotentials and (c) Tafel plots, and (d) Nyquist plots of the electrocatalysts during HER activity in 1.0 M KOH.

Table S1: Textual properties of pristine MoS_2 and the MoS_2@rGO hybrid samples

Sample	BET surface area (m^2/g)	Monolayer Pore volume (cm^3/g)	Pore vol. (cm^3/g)
**MoS_2**	45.5	10.4	0.2936
**MoS_2@rGO**	56.1	12.9	0.2698
**rGO**	185.8	42.7	0.7747

Table S2: Raman parameters for the pristine MoS_2 and the MoS_2@rGO hybrid samples

Sample	Peak position (cm^{-1})				Defect density ratio (I_D/I_G)	Δ (A_{1g}-E_{2g})
	E_{2g}	A_{1g}	D-band	G-band		
**MoS_2**	381.4	406.1	-	-	-	24.7
**MoS_2@rGO**	380.6	406.4	1345.8	1587.7	3.17	25.8

Table S3: Atomic compositions of the composite samples

Samples	Elements (at. %)				
	C 1s	N 1s	O 1s	Mo 3d	S 2p
**MoS_2**	39.41	14.87	2.88	17.24	25.61
**MoS_2@rGO**	56.72	6.77	5.15	12.08	19.29

Table S4: EIS analysis of pristine MoS_2 and the MoS_2@rGO hybrid samples post HER experiments

Sample	R_s (Ω)	R_{ct1} (Ω)	R_{ct2} (Ω)
**MoS_2**	9.94	560	-
**MoS_2@rGO**	16.9	74.3	30.4
